# Why so Complex? The Intricacy of Genome Structure and Gene Expression, Associated with Angiosperm Mitochondria, May Relate to the Regulation of Embryo Quiescence or Dormancy—Intrinsic Blocks to Early Plant Life

**DOI:** 10.3390/plants9050598

**Published:** 2020-05-08

**Authors:** Corinne Best, Ron Mizrahi, Oren Ostersetzer-Biran

**Affiliations:** Department of Plant and Environmental Sciences, The Alexander Silberman Institute of Life Sciences, The Hebrew University of Jerusalem, Edmond J. Safra Campus—Givat Ram, Jerusalem 9190401, Israel; corinne.salmanlevi@mail.huji.ac.il (C.B.); ron.mizrahi1@mail.huji.ac.il (R.M.)

**Keywords:** plant, mitochondria, posttranscription, RNA-metabolism, editing, splicing

## Abstract

Mitochondria play key roles in cellular-energy metabolism and are vital for plant-life, such as for successful germination and early-seedling establishment. Most mitochondria contain their own genetic system (mtDNA, mitogenome), with an intrinsic protein-synthesis machinery. Although the challenges of maintaining prokaryotic-type structures and functions are common to Eukarya, land plants possess some of the most complex organelle composition of all known organisms. Angiosperms mtDNAs are characteristically the largest and least gene-dense among the eukaryotes. They often contain highly-variable intergenic regions of endogenous or foreign origins and undergo frequent recombination events, which result in different mtDNA configurations, even between closely-related species. The expression of the mitogenome in angiosperms involves extensive mtRNA processing steps, including numerous editing and splicing events. *Why do land-plant’s mitochondria have to be so complex*? The answer to this remains a matter of speculation. We propose that this complexity may have arisen throughout the terrestrialization of plants, as a means to control embryonic mitochondrial functions —a critical adaptive trait to optimize seed germination. The unique characteristics of plant mtDNA may play pivotal roles in the nuclear-regulation of organellar biogenesis and metabolism, possibly to control embryos quiescence or dormancy, essential determinants for the establishment of viable plantlets that can survive post-germination.

## 1. Overview

The evolution of land plants from a green algal ancestor, about half a billion years ago [1,2], was a remarkable event in the history of life on earth. The transition from aquatic to terrestrial life was associated in plants with the development of different genetic and physiological mechanisms, allowing them to cope and adapt to various environmental stresses, such as high light intensities, UV radiation, fluctuating temperatures, salinity, limited water supply and drought [3,4,5,6]. These included the development of specialized structures and new reproductive strategies, as well as many adaptations in cellular metabolism. One of the most notable adaptations that enabled the establishment of Spermatophytes, which include gymnosperms (conifers, cycads and ginkgos) and angiosperms (after “seed within a vessel”[7], also termed as flowering plants) on land involves the production of highly sophisticated dispersal units, known as seeds [8]. In this article, we discuss the main characteristics of plant mtDNA structures and gene expression patterns, with an emphasis on mtRNA metabolism, and their significance for seed and embryo development, germination and the establishment of viable plantlets that can survive post-germination.

The seed is a critical stage in the life cycle of flowering plants (see Section 2, below), with respect to their survival as a species [9], and its evolution represents a crucial feature that allows the plant embryos to survive in the period between seed maturation and dispersal to the next generation as a seedling (after a successful germination). The development and maturation of seeds requires elevated cellular energy metabolism and mitochondria-related functions (see Section 3, below) [10,11,12]. These processes depend on a tight regulation on mitochondrial activities [13]. Many seeds enter a quiescence or dormant periods (see Section 2.2, below), in which cellular metabolism and respiration are greatly reduced to specifically allow germination under favorable environmental conditions. One of the earliest events of seed germination is a progressive proliferation and/or differentiation of mitochondria, cellular functions known as ‘mitochondrial biogenesis’ [11,12,14,15]. These processes rely on a complex nuclear-organellar cross talk and the communication of mitochondrial status with cellular networks. Angiosperm mitochondrial genomes (mtDNAs, mitogenomes) are undoubtedly the most complex among the eukaryotes. The expression of their mtDNAs is regulated mainly at the posttranscriptional level, as evident by the extensive mtRNA processing events, for example, trimming, editing, and splicing [13]. It remains unclear, however, why plants have acquired such complex mechanisms to regulate the expression of mitochondrial genes. A hypothesis we raise here is that the introduction of new levels of ‘molecular-maturation’ provided the land plants with improved regulation of organellar functions.

From a botanical point of view, functional and molecular analysis of angiosperm mitochondria provide important insights into the evolution of land plants, nuclear-cytoplasmic interactions, and mitochondria-related physiological traits [16]. These features are expected to be highly valuable for future agriculture and crop improvement, especially under suboptimal growth conditions. Our focus for this manuscript is studies aimed at understanding the evolutionary selection pressures that have shaped the angiosperms mitogenome organization and the intricacy of their gene expression designs. We speculate that this organellar complexity relates to the necessity of land plants to regulate germination and to maintain that their offspring are quiescent or dormant until environmental conditions become favorable for their germination.

## 2. Embryogenesis, Seed Maturation, and Germination Rely on Mitochondria Functions and Cellular Metabolism

### 2.1. Seed as a Major Adaptation of Plants to Life on Land

Seeds consist of ‘a miniature undeveloped plant’ (i.e., the embryo) and nutrients stored in specialized seed tissue (endosperm) or within the embryo itself. The evolution of seeds represents an exceptional transition for plants that invaded the land. The kingdom ‘Plantae’ constitutes a large and varied group of organisms, with more than 300,000 species [17], about 90% of these are seed plants. Fossilized samples of early land plants can be dated as early as the Ordovician period [18]. During the evolution, land plants have acquired various mechanisms that allow them to escape or adapt to a wide range of growth conditions. Bryophytes, which include liverworts, hornworts, and mosses, are regarded as the most basal group among land plants. As their proposed algal predecessors [19], bryophytes remained largely dependent on water for survival and reproduction [1]. A major issue to life on land involves desiccation tolerance. Accordingly, many mosses are able to dehydrate until water becomes available. Angiosperms developed additional strategies and new structures, which allowed them to withstand dry environments rather than to tolerate them, as some bryophytes [1]. These include the development of complex fruit organs, and the dispersal of durable seeds, to ensure the survival of their offspring.

The seeds of land plants harbor a diploid embryo that germinates into a sporophyte [20]. The sporophytes of early land plants gradually gained independence by evolving photosynthetic and vascular tissues [21]. Photosynthetic sporophytes of some mosses are dependent on the gametophyte for nutrients, further supporting this theory [22]. Meiosis in the reproductive organs of plants leads to the production of haploid male and female gametophytes, i.e., pollen grains in the anthers and egg cells in the ovules. During fertilization and following the attachment of the male gametophyte to the stigma, a pollen tube elongates through the megasporangium wall for the delivery of the sperm cells to the female gametophyte (for a recent review, see, e.g., [20]). The fertilization of the egg leads to the formation of a diploid zygote that starts to develop into an embryo within the emerging seed. In different plants, multiple fertilizations are accompanied by maturation of the ovary into a complex fruit organ. The ripening of fruits, which is synchronized with seed maturation, is a tightly regulated genetic process [23], which ranges from dry organs, as pods or siliques, into fleshy fruits where the pericarp and accessory parts develop into succulent tissues (reviewed by, e.g., [24]). The dry pods or siliques contain mature seeds that are often dispersed by a physical force. These have been presumably evolved earlier than their fleshy organs counterparts, which typically rely on animals that eat the fruit and disperse the seeds after ingesting or discarding them [25]. The mature seeds of land plants show a huge diversity in shape, size, and internal architecture, which relate to their dispersal characteristics and for protecting the embryos from both abiotic and biotic cues [26,27]. During the maturation, the seeds accumulate high levels of nutrients that are stored in the seed endosperm (often starch and protein) or within the embryo’s tissues (mainly lipids), in order to sustain cellular activities in the embryo, and mostly for the high-energy requirements during germination and the establishment of the young plantlets [23,28,29].

### 2.2. Seed Quiescence, Dormancy, and Germination

Germination is an essential determinant in early-seedling development [8,9,30,31,32]. The timing of germination is critical and is tightly correlated with seedling survival rates, vegetative growth, and reproduction. All major crops require intensive breeding programs, where the elite cultivars are distributed in the form of seeds. Ensuring high germination rates is, therefore, critical for agricultural production, and thus for our future food security. Many seeds have developed a rigid and waxy protective coat that protects them from the environment and provides them with a superior evolutionary advantage to life on land [30]. The dispersal of seeds in many plant species occurs during the dry season, but they can remain viable for prolonged periods of time, especially when they are desiccated [33]. An important strategy in seed-plant physiology involves dormancy, an intrinsic block to early-plant development that allows the embryos to survive under unfavorable environmental conditions [9,31,34,35]. Dormancy is enforced in seeds via various factors, as desiccation, the toughness of the seed coat (prevents water-uptake and limits air supply), or by endogenous biochemicals that inhibit embryo development. An important feature of dormancy involves the inhibition of simultaneous germination of the seeds [33]. This phenomenon is of mechanistic interest, but at the same time represents a problem to the agricultural industry that relies on uniformity [31]. Another proposed mechanism to postpone germination is ‘seed quiescence’ [9,31,34]. As dormant seeds, quiescent embryos maintain low metabolism until environmental factors necessary for germination (e.g., water, heat, light, and oxygen) are perceived. Generally, seeds that are in an environment optimal for germination but fail to complete germination are regarded as dormant [35]. The germination of quiescent or dormant seeds is determined by an interplay of various factors, such as light, temperature, water, nutrients, mechanical forces, and hormonal signals. This ensures that germination would occur specifically when the seed perceives a combination of environmental signals necessary for successful seedling emergence and establishment. As soon as the seeds absorb water (a key stage termed as imbibition), a notable burst in respiration occurs, in order to generate the required energy to power germination and early-seedling establishment.

Germination is a high-energy consuming process that necessitates functioning mitochondria immediately following seed imbibition [11,12,14,15,36], and is generally divided into three main phases: (*i*) imbibition, (*ii*) reactivation of metabolism, and (*iii*) radicle protrusion [31]. Enzymes of the glycolytic, pentose phosphate, amino acid metabolism, and the tricarboxylic acid (TCA, Krebs cycle) pathways play key roles during embryo development and germination [37]. The most critical stage seems to be the metabolic phase, where a rapid increase in enzymatic hydrolysis of food reserves (proteins, lipids, and carbohydrates) and elevated respiration provide the necessary energy supply for the initiation of seed germination (see, e.g., [14,29,31,38]). The initial breakdown of stored nutrients at the onset of germination is commonly considered to be facilitated, mainly, by ‘anaerobic respiration’ [39]. According to this model, enzymes such as lactate dehydrogenase or succinate dehydrogenase mediate the metabolism of storage lipids and carbohydrates under low oxygen levels. These pathways allow the generation of low levels of ATP, whereas the OXPHOS system allows the complete metabolism of carbohydrates into CO_2_, with the concomitant production of large amounts of ATP in the cell, about 15× higher than the glycolytic process. These activities rely on a tight regulation on mitochondria biogenesis and organellar gene expression (see Section 3.3, below).

Some seeds demonstrate remarkable viability periods, as the ~1300 year-old seeds of Lotus (*Nelumbo nucifera*) recovered from a dry lakebed in northeastern China [40], ancient date-palm (*Phoenix dactylifera*) seeds found in Masada, Israel, which were able to germinate after ~2000 years and to produce viable trees [41], or the extreme case of *Silene stenophylla* in which their seeds remained buried for ~32,000 years in the Siberian permafrost and yet were able to regenerate into fertile plants [42]. How the embryos can remain viable for such long periods? All living forms need to maintain basic cellular functions, where they consume nutrients and use energy to carry out the chemical reactions that sustain life. Assumingly, without controlling their organelles, the embryos would eventually consume the seed reserves, and thus, fail to germinate or to produce viable seedlings when the right time to sprout has come. We speculate that large organellar genomes with a complex mode of gene expression, in particular at the posttranscriptional level, allow plants to tightly control the respiratory-mediated functions, and thus to minimize cellular metabolism, until environmental conditions are favorable for germination. The organellar characteristics that enable land plants to maintain seed viability on one side, while on the other side allow them to regulate germination by controlling mitochondrial biogenesis, gene expression, and function, are discussed below.

## 3. Land Plant Mitochondria Genome Structures and Gene Expression, and Their Essential Roles in Successful Germination and Early Seedling Establishment

### 3.1. Mitochondria Biogenesis and Respiratory Reactivation during Seed Germination

Mitochondria, which house the oxidative phosphorylation (OXPHOS) machinery, the TCA cycle, as well as numerous other essential metabolic pathways in plants [43,44], play key roles during germination, early seedlings establishment, and at later developmental stages [12,15,28,45]. These organelles may be viewed as “wild and selfish” prokaryotic-type structures, which need to be “tamed” by the hosts. Also, as each organelle acts independently, in a semi-autonomous manner, mitochondrial activities need to be synchronized and optimized with the cell metabolic needs. Accordingly, the biogenesis and functions of the energy-producing organelles is a tightly regulated, multi-step process that relies on the coordination of many organellar processes, as mtDNA replication, transcription, mtRNA processing and maturation, translation, and the assembly of organellar complexes, which contain both nuclear and mitochondria encoded subunits [13].

Studies focusing on mitochondria biology in different angiosperms pointed to novel, but at the same time conflicting, characteristics of mitochondrial biogenesis during the rapid development of germinating embryos [8,9,11,12,15,31,33,38,45,46,47,48,49]. Two main models for plant mitochondria biogenesis have been proposed: (a) the activation of mitochondria that are already present in the seed, and (b) the maturation of mitochondria from pro-organelles found in the embryonic tissues. The first model describes the biogenesis of fully active mitochondria by growth and division (fission and fusion) processes from pre-existing organelles, whereas according to the alternative (second) model, mitochondria differentiate from non- or partially-functioning pro-mitochondria found in the seeds.

Early studies have indicated that elevated respiration is accomplished by the activation or synthesis of various mitochondrial enzymes required in the electron transport chain and the TCA cycle [33]. In dry seeds, mitochondria do not have the characteristic internal membrane system (i.e., cristae) [15,47], which is regarded as critical for the assembly of the respiratory chain system and the ATP-synthases enzymes [50]. As the levels of O_2_-uptake rates of embryos found in dormant or quiescent cells are often very low, or below detectable levels, it was anticipated that during seed maturation the mitochondria dedifferentiate into non-functional organellar forms. Yet, studies of mitochondria biogenesis of two grass species, maize (*Zea mays*) and rice (*Oryza sativa*), suggested that the embryos found in the dormant or quiescent seeds harbor pro-mitochondria-like structures that are already active, and which are fueled by external NADH [15,47,51]. In support of these observations, blue-native gel electrophoresis (BN-PAGE) indicated that components of the mitochondrial electron transport system are already present in the dry seeds of Arabidopsis, and gradually increased in their abundances during the imbibition, showing the highest abundances in mature seedlings [52]. Live imaging of mitochondria membrane potential of Arabidopsis embryos further indicated that organellar bioenergetic reactivation occurs immediately upon rehydration [14] and suggested that mitochondria remain suppressed until the embryos perceive the appropriate signals for germination. Together, these data are supporting a pro-mitochondria differentiation model.

The characteristics of plant mtDNA structures and gene expression may play key roles in the nuclear-control of organellar biogenesis during seed development, in order to maintain embryos quiescence or dormancy. Although seeds provide a high level of endurance, even under optimal storage conditions their viability decrease over time, a phenomenon of great impact on seedlings development and productivity, and also a cause for commercial and genetic losses [10]. We propose that complex mitogenomes with a highly regulated mode of gene expression may provide the angiosperms with an improved means to control their organellar functions, in order to regulate developmental arrest, and when required to allow rapid changes in cellular metabolism to support the high-energy demand for the developing embryos during early germination.

### 3.2. Organization of Angiosperm Mitochondrial Genomes

The acquisition of mitochondria is a hallmark in the evolution of eukaryotes. About 140 years ago, Andreas F.W. Schimper noticed that chloroplasts share similarities with cyanobacteria [53]. Konstantin Mereschkowski, which was familiar with the work, suggested that eukaryotic cells have originated through a symbiosis between “separate, single-celled organisms”[54], about 1.5 billion years ago [55]. Concurrently, Carl Correns and Erwin Baur reported the ‘non-Mendelian’ inheritance features of chlorophyll deficiencies in the plants Pelargonium and Mirabilis [56,57]. These findings founded the field of ‘extranuclear genetics’ [58], which was later supported by studies with *Saccharomyces cerevisiae* (i.e., ‘petite’ mutants), which indicated the existence of mtDNA as the heritable cytoplasmic element of yeast cells [59]. The endosymbiotic theory was advanced and substantiated with microbiological evidence by Lynn Margulis [60]. It is widely accepted that mitochondria have originated from a single endosymbiosis event, involving a proteobacteria-like organism and the common cellular ancestor of eukaryotes [61]. Yet, during the evolution, the mtDNAs have diverged considerably among different eukaryotic species. Currently, there are ~11,000 eukaryote mitogenome sequences available at the ‘National Center for Biotechnology Information’ (NCBI). Data about the composition of different mitogenomes from various organisms, including Plant, Fungi, animals, and the genome of Rickettsia (the proposed α-proteobacterial ancestor of mitochondria), are shown in Table 1.

Since the early investigations of angiosperms mtDNAs, using electron microscopy and gel electrophoresis [62], a wealth of information has been subsequently accumulated (for recent reviews see, e.g., [63,64]). The first complete genome of *Arabidopsis thaliana* was described about 20 years ago [65], with its mtDNA (~370 kb, Table 1) completely sequenced in 1997 [66]. The mtDNAs of angiosperms are notably the largest known to date (see Table 1), some of which are larger than bacterial genomes or the nuclear DNA in some eukaryotes (e.g., the 11.3 Mb mtDNA in *Silene conica*) [67]. For instance, the size of mtDNA in the ancient angiosperm *Amborella trichopoda* (i.e., 3866 kb) [68] is about 200 times larger than the mitogenomes found in animal cells (i.e., 15~20 kb; Table 1).

The increased size of the mtDNAs in land plants (see, e.g., [63,64,67,72,73,74]), relative to their algal ancestors, is accounted mainly for the expansion of intergenic DNA regions. These regions contain sequences that are key to mtDNA expression, for example, promoters, ribosome binding sites, UTR regions, and intervening intron (mostly group II-type) sequences [13]. Gymnosperms [74] and some embryophyta species [75] also have very large mitogenomes, in which the sizes may exceed a million base pairs. The mtDNAs of land plants often exhibit variable quantities of foreign sequences of plastid or nuclear origins [76], and also seem to undergo frequent recombination events between different repeated sequences scattered throughout the mtDNAs, which result with different mitogenome configurations, even between closely-related plant species [63,64]. While mitogenomes are commonly described as circular DNA structures, studies in land plants generally failed to recover a single circular mtDNA molecule [77], termed as the ‘master circle’. Mitochondria of angiosperms may harbor linear, branched or multichromosomal (linear or circular) DNA structures (see, e.g., [78]). Some *Silene* species can contain as many as 50 mtDNA molecules, which evolve fast by a gain or loss of different molecules due to recombination events [67]. Recombination within angiosperm mitochondria also leads to the generation of chimeric open reading frames (ORFs), some of which are associated with cytoplasmic male sterility (CMS) [79,80].

Despite the large variations in mitogenome sizes and gene organization, the number of known mitochondrial genes (between 60 to 70) is relatively conserved among different terrestrial plant species [63,64]. Typically, the mtDNA gene content of early land plants is also reduced compared with Spermatophytes [81,82,83], with an exception of the mistletoe mitogenome, which has undergone massive gene losses [84,85,86]. In some species, as *Amoebophrya ceratii*, the entire mitogenome has been translocated into the nuclear genome [87], whereas various anaerobic organisms have lost the respiratory activities [88]. If organellar genes can be mobile, why have most mitochondria (and plastids) retained their genomes, and why do land plants possess such large and complex mtDNA configurations? One hypothesis is that expression of genes encoding core OXPHOS subunits remained within the organelle to facilitate redox regulation [89]. Computational modeling, using 2000 different mtDNAs from animals, fungi, protists and plants, indicated that genes encoding proteins of the respiratory or ribosomal complexes are more likely to be retained in the mitogenome [90]. The authors suggested that keeping these genes in the mtDNA provide the cell with means to individually control mitochondria biogenesis. Here, we further speculate that land plants, and angiosperms in particular, have evolved large and complex mtDNAs to regulate seed quiescence and dormancy and to control germination.

### 3.3. Plant Mitochondria Gene Expression, and Its Regulation during Early Germination Stages

The coordination of growth and development is achieved by cellular signaling, allowing plants to regulate and coordinate their energy demands during particular growth and developmental stages. A complex network of genetic interactions between the organelles and the nucleus regulates the metabolic functions, biogenesis, and maintenance of the mitochondria. The mitochondrial ribosomes and the energy transduction machineries are assemblies of both nuclear and organellar encoded subunits. The correct stoichiometry in the accumulation of the different subunits composing the organellar complexes is, therefore, essential for mitochondrial biogenesis, and hence for respiratory functions [91]. These processes rely on complex mechanisms for regulating the coordination of the expression and accumulation of the different subunits encoded by the physically remote genomes, involving both anterograde (nucleus to organelle) and retrograde (organelle to nucleus) signaling [92,93].

**Figure 1:**
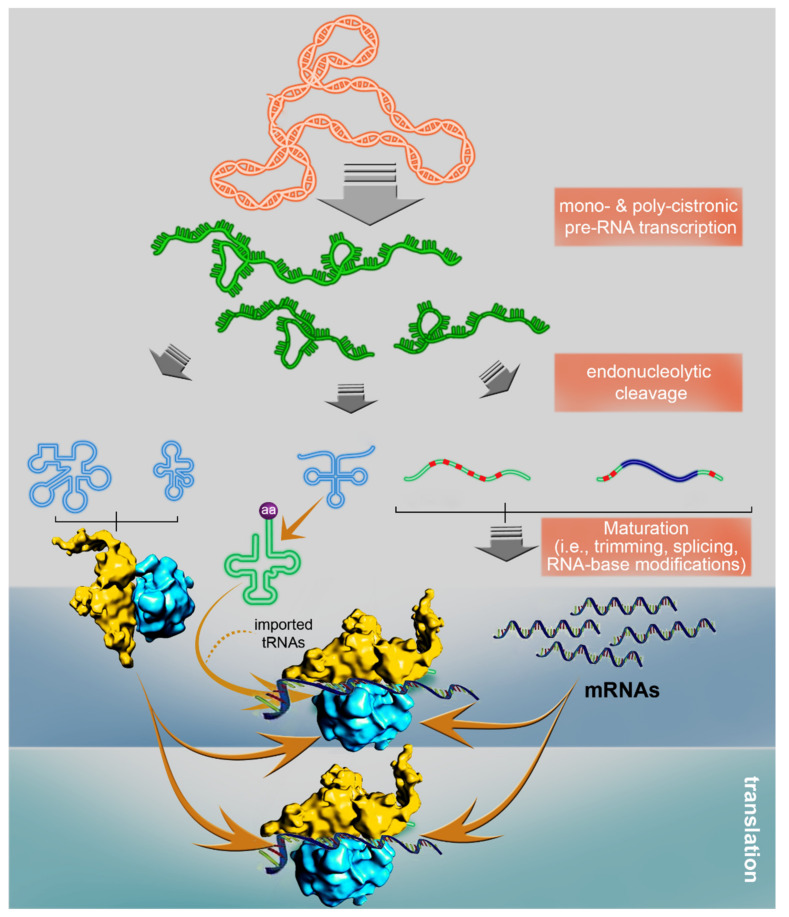
Mitochondrial gene expression in angiosperms. The mitogenome harbors tRNAs, rRNAs, and various protein-coding genes. The flow of information from the mtDNA in flowering plants to the translation of the mRNAs involves numerous posttranscriptional steps. These include endonucleolytic processing of polycistronic transcripts, maturation of 5’ and 3’ termini, extensive RNA editing (red boxes) and group II intron splicing (marked in blue). Translation is initiated by the ribosomes assembled on the mRNAs, with organellar-encoded and imported tRNAs participating in the elongation of the polypeptide. The structure of Arabidopsis mitochondria ribosomes was modified from [97].

Among the many different eukaryotic organisms examined to date, land plants are found to contain the largest mitochondrial genomes. However, the size of the mtDNA is not the only characteristic that makes land plant mitochondria truly exceptional. The RNA metabolism characteristics of plant mitochondria (see Figure 1) are strikingly different from those of their prokaryotic ancestors and corresponding organelles found in Animalia. The mitochondria of flowering plants are, in particular, rich in introns (mostly group II-type), some of which are independently-transcribed as separate intron segments that are spliced in “trans” [94,95,96], and many of the primary mtRNAs must be modified by RNA editing in order to become functional transcripts, where these (and other) posttranscriptional processing events have the predominant role in determining gene product abundance [13]. The main processing steps of transcription, pre-RNA processing and translation in plant mitochondria are presented in Figure 1.

Comparing the sequences of mtRNAs to their corresponding organellar gene-loci, it became apparent that the expression of the mitogenomes in plants is extremely complex, particularly at the posttranscriptional level [66]. In animals, the entire mtDNA is transcribed from two initiation sites, resulting in two long polycistronic pre-RNAs denoted as the light (L) and heavy (H) strands [98]. In contrast, the physical arrangement of the genes within the mtDNAs in land plants indicates that numerous transcription initiation sites are required for the expression of the complete set of mitochondrial genes [13]. The primary polycistronic transcripts (pre-RNAs) in angiosperm mitochondria then undergo extensive processing steps [13], which include the maturation of 5’ and 3’ termini, RNA editing [99] and intron splicing. These processes are essential for the organellar RNAs to carry out their functions in protein synthesis and are mediated by a large number of nuclearly encoded proteins that are imported to the mitochondria (see Section 3.3.1) and may also serve as key control points in plant mtDNA expression. Many of these factors are specifically expressed during the earliest stages in germination and likely regulate early plant development [48,49].

#### 3.3.1. The Biogenesis of the Import Machinery: A Molecular Switch for RNA Metabolism and Mitochondria Biogenesis during Early Germination

Although mitochondria contain their own genetic system, which is separate and distinct from the nuclear genome, they are not self-supporting entities and rely on imported nuclear gene products for their proper functions. In addition to the several dozens of organellar-encoded proteins, the mitochondria also host numerous nuclear-encoded proteins that play indispensable roles in respiration-mediated activities, in regulation of mtDNA expression, RNA processing, translation, and the metabolism of the OXPHOS system. Recent analysis of the Arabidopsis mitochondria proteome indicates to nearly 1000 different proteins [100]. The majority of the host-encoded proteins are synthesized as ‘precursor proteins’, containing amino-terminal extensions, which are proteolytically cleaved to form the mature functional organellar proteins [101]. Their insertion into the mitochondria is facilitated by a molecular import machinery, known as the TOM-TIM23 [102,103]. The biogenesis of the mitochondrial import system of plants seems to be regulated during embryo maturation and seed germination. A notable decline in the levels of various TOM and TIM subunits is evident in the mitochondria of mature seeds [47], whereas the expression of several TOM and TIM subunits was found to be notably upregulated in the seeds during early stratification, compared to dry seed [28].

The biogenesis of the TIM-TOM system is a prerequisite for the import of host-encoded protein cofactors that function in the expression of the mtDNA, at the earliest stages in germination. The metabolism of mtRNAs is accomplished, largely, by different nuclear-encoded RNA binding proteins, which may also provide a means to link organellar functions with developmental or environmental signals [13,104]. Results obtained from transcriptomic data indicate substantial increases in mitochondrial transcript levels during the first three hours of seed imbibition [28,49]. A notable pick in the expression of proteins associated with RNA editing and group II intron splicing (see below) precede the upregulated expression of mitochondrial genes encoding subunits of the OXPHOS system soon after the accumulation of imported related subunits (and, thus, the biogenesis of the TIM-TOM machinery) [49]. These data strongly suggest that the regulation of mtRNA processing plays a pivotal role in plant mitochondria biogenesis during seed germination and early plantlets development. Below, we discuss some of the principle components of the posttranscriptional regulatory steps in plant organelle gene expression (Figure 1), and their putative roles in regulating seed germination and early seedling establishment.

#### 3.3.2. RNA Editing Plays a Key Role in the Regulation of mtDNA Expression

The term RNA editing was first coined in the 1980’s, when researchers have realized that Uridine nucleotides are inserted posttranscriptionally into mRNAs in *Trypanosoma brucei* mitochondria [105]. Posttranscriptional RNA base exchanges in land plant mitochondria were recognized shortly after the discovery of RNA editing in trypanosomes [106,107,108]. For a recent review on plant mtRNA editing, see [99]. In flowering plants, RNA editing mainly involves C-to-U substitutions, while reverse U→C editing is shown in basal plants [109,110,111,112]. Biochemical analyses further indicates that many transcripts in angiosperms mitochondria undergo many *N^6^*-methyladenosine (m^6^A) modifications [113,114], and that m^6^A may affect the expression of various mitochondrial proteins [114]. Differently from m^6^A modifications, RNA editing has not been detected yet in bacteria or algae (with the exception of an assumed single editing event in *trnI-CAU* of *C. pacifica* [115]) (see Table 1), suggesting that it has been evolved in plant organelles only after the terrestrialization of plants, about half a billion years ago.

In plant mitochondria, RNA editing often occurs at the first or second base of a codon, where many of these events restore codons that encode amino acids that are conserved in evolution [107]. A possible role for editing in the regulation of plant mtDNA expression has been suggested previously [116,117], although this is still under debate. Variable RNA editing frequencies are observed at cryptic translation initiation sites or termination codons, the 5′ or 3′ UTRs of some mtRNAs, in tRNAs and rRNAs, as well as in sequences corresponding to group II introns [99]. Differences in RNA editing profiles in different tissues or under various developmental conditions have been previously reported [118,119,120], while some mutants affected in mitochondrial editing show embryogenesis defect phenotypes [121,122]. Since the initial discovery of RNA editing in plant mitochondria [106,107,108], the enzymatic properties and specificities of the processes have been intensively investigated. Genetic analyses of mutants affected in mtRNA editing indicate that pentatricopeptide repeat (PPR) proteins, first described in *A. thaliana* [123], serve as the primary recognition factors that single out specific cytidines to be converted into uridines in plant organellar genomes [99]. Notably, the PPRs also provide the cytidine deaminase (or DYW domain, named for the presence of conserved Asp–Tyr–Trp residues in the C-terminus) that carries out the nucleotide modification reaction [124]. Some PPRs lack the DYW domain. These recognize the specific editing site, and recruit DYW-PPRs to facilitate the deamination of the C residue [99]. Transcriptome analyses indicate that many of the 261 PPR proteins, known or predicted to the mitochondria of Arabidopsis plants, showed transient expression, peaking in expression following 48 hours of seed stratification [49]. These data further indicate the importance of mtRNA editing during embryo development and at early stages in seed germination.

#### 3.3.3. Presence and Putative Role of Group II-Type Introns in Angiosperm Mitochondria

One of the most remarkable features of the mitogenomes in land plants involves the presence of many group II- intron sequences [94,125], which reside within the coding regions of many essential organellar genes [13,95,96,126]. Group II’s are a class of self-catalytic RNAs (ribozymes) and mobile genetic elements, reminiscent of the ancient RNA world, which have invaded into the genomes of prokaryotic organisms and the organellar genomes of some eukaryotic organisms [127,128,129]. Genomic analyses indicate that the common ancestor of green algae and land plants most likely harbored a tightly packed, gene-rich, and intron-poor mitogenome [63,64,67,73,130,131]. The number of introns in the mtDNAs of land plants was greatly increased, with more than 20 group II introns (Table 1), which reside in protein encoding genes, mostly within NADH dehydrogenase (complex I) subunits [13,64]. These have likely spread to new mtDNA sites during the evolution of charalean green algae and bryophytes, accounting for part of the intron diversity found in Chara and land plant mitogenomes [71]. Streptophyte green algae, which live in freshwater, are able to tolerate conditions that resemble drought, a condition that is forced by increased salinity during water evaporation at particular times [132]. Freshwater adaptation by the green algae species may have allowed a gradual move of the early land plants towards moist habitats in the proximity of water, and ultimately the colonization of dry land dependent on rainwater [132]. An intriguing possibility is that land plants, as their Charales predecessor, have acquired group II introns in their mitochondria to better control their organellar gene expression, as a means to cope with similar challenges in their lifestyle, in particular, during germination and the establishment of young plantlets on land.

Canonical group II introns consist of an autocatalytic RNA and a protein component, i.e., a retroviral reverse-transcriptase (RT) like protein, known as a maturase [13,96,127,128,129]. Throughout the evolution, the organellar introns in land plants have diverged considerably from their bacterial ancestors, such as they have lost many elements that are considered to be essential for self-splicing and also lost the vast majority of the intronic ORFs encoding for maturases [94,125]. For their splicing, the group II introns in angiosperm mitochondria rely on nuclear encoded protein cofactors. The evolutionary dependence of angiosperms’ mitochondrial gene expression on host-encoded factors is further reflected by the unusual organization of several mitochondrial genes as fragmented transcripts separated by group II intron sequences, which are individually transcribed at different loci in the mtDNA (reviewed by, e.g., [13,95,96,126]). The formation of a functional mRNA, thus, relies on the assembly of different intron pieces to form a splicing-competent structure in a process termed ‘trans-splicing’ [94]. Genetic studies have established the roles of numerous nuclear encoded proteins in the splicing of different subsets of mitochondrial group II introns [13,95,96]. The majority of these proteins are not found among the different proteins identified in global mass-spectrometry analyses of plant mitochondrial fractions, presumably, as these are very lowly expressed in the vegetative tissues. Microarray and RNA-seq data indicate that many of the factors show differential expression, where they exhibit dominant expression in embryonic tissues and accumulate to high levels particularly during imbibition and early germination stages [48,49]. Moreover, temperature- and tissue-depended splicing have been shown for mtRNAs during germination and early seedling establishment [46,133,134], further signifying the importance of group II intron splicing as a means to regulate the expression of mitochondrial genes during these critical stages in land plant life.

#### 3.3.4. A Mitochondrial Failure in Angiosperms Often Results in Altered Embryogenesis, Reduced Germination, and Retarded Seedlings Growth and Development

The maturation (e.g., RNA editing and splicing) of mitochondrial transcripts in land plants is critical for the expression of the genes they interrupt, and hence for respiratory functions and plant development. The impact of mtRNA metabolism defects on embryogenesis (or gametogenesis) is now becoming increasingly evident in land plants (see, e.g., [135]). The function of many nuclear encoded RNA editing and splicing factors was found to be critical during embryogenesis, seed maturation and germination, or during early plantlets development. Accordingly, mutations that severely affect the biogenesis of the respiratory system, or other aspects of organellar activities, such as a disruption in the organellar translation machinery, often result in embryo developmental arrested or lethality phenotypes (see, e.g., [91,135,136,137,138,139,140,141,142,143,144]).

#### 3.3.5. Angiosperm Mitochondria Gene Copy Numbers

Mitochondria in yeast cells contain between 50 to 200 mtDNA copies, and 1 to 10 copies of mtDNA per mitochondrion in animals. Yet, in Arabidopsis there is an estimate of only 50 copies of mitogenome per cell [145], suggesting that less than 10% of the mitochondria contain DNA. These data are also in agreement with earlier observations suggesting that many cucurbit mitochondria lack DNA [146]. The absence of mtDNA in many of the organelles may relate to the massive and frequent fissions and fusions cycles characteristic to plant mitochondria and may explain how mitochondrial genomic information is shared within plant cells [147]. It would be interesting to examine whether plant mitochondria utilize RNA as their main genetic material, and whether RNA processing and translation are developmentally regulated, for example, during germination and early seedling establishment.

## 4. Concluding Remarks

Land plants possess the most complex organelle composition of all known organisms. While mitochondria found in the embryonic cells of animals remain fully active throughout development, the mitochondria of embryos in flowering plants undergo a (limited) dedifferentiation during seed maturation. Mitochondria in mature dry seeds maintain only basal cellular activities, whereas seed awakening requires a rapid increase in cellular metabolism that is critical for the establishment of the young seedlings. These processes are accompanied by a notable burst in respiratory functions. Plant mitochondria biogenesis is tightly regulated during seed development and germination. During 120~170 million years of evolution [148,149], angiosperms have acquired a huge set of protein cofactors, harboring a variety of RNA binding modules, to manage the specificity of plentiful different mtRNA metabolism reactions [13]. Recent studies suggest that RNA processing enzymes comprise a staggering proportion of the total proteome of angiosperm mitochondria (i.e., 14.9%), further signifying the importance of mtRNA metabolism for organellar biogenesis and plant physiology [100]. Many of the mtRNA processing factors appear to be notably upregulated during germination and early seedling development. The translation of the mature organellar transcripts also seems to be under cellular control [97,150]. A hypothesis we raise here is that the introduction of additional levels of organellar ‘molecular maturation’ (and translation), during evolution in land plants, allows an improved control of respiratory functions during maturation, to maintain seed quiescence or dormancy and to allow rapid changes in cellular metabolism for the developing embryo. An intriguing possibility is that the increased complexity of mtDNAs within the cells of fungi and protists (Table 1) may relate to the necessity of these species to also withstand harsh environmental conditions during their lifetime.

## Figures and Tables

**Table 1:** Mitochondria genome structures in different classes.

Phylogeny		Organism	mtDNA Size (Kb)	Accession No.	C→U Editing	Group I Introns^*1^	Group II Introns
Bacteria	Rickettsia^*2^	*Rickettsia proazekii*	(1109.3)	NC_020993	-	-	-
Plantae	Green Alga	*Caulerpa ashmeadii*	197.4	NC-045849	-	-	-
		*Chara vulgaris*	67.7	NC-005255	-	13	14
		*Chlamydomonas reinhardtii*	15.8	NC-001638	-	-	-
		*Chloroparvula pacifica*	49.7	NC-042603	(?)^*3^	2	-
	Land Plants^*4^	*Arabidopsis thaliana^A^*	367.8	NC-037304	+	-	23
		*Climacium americanum^B^*	105.1	NC_024515	+	2	23
		*Brassica oleracea* (Cauliflower)^*A*^	360.2	NC-016118	+	-	23
		*Ginkgo biloba^G^*	346.5	NC_027976	+	-	20
		*Oryza sativa* (Rice)^*A*^	491.5	NC_007886	+	-	25
		*Silene latifolia^A^*	253.4	NC_014487	+	-	13
		*Triticum aestivum* (Wheat)*^A^*	452.5	NC-036024	+	-	23
		*Welwitschia mirabilis^G^*	978.9	NC_029130	+	-	17
		*Zea mays (Corn)^A^*	569.6	NC-007982	+	-	22
Fungi		*Pichia canadensis*	27.7	NC-001762	-	2	-
		*Saccharomyces cerevisiae*	78.9	NC-027264	-	3	9
		*Schizosaccharomyces pombe*	19.4	NC-001326	-	2	1
Animalia	Insects	*Anopheles gambiae (Mosquito)*	15.4	NC-002084	-	-	-
		*Apis mellifera (Honeybee)*	16.3	NC-001566	-	-	-
		*Drosophila melanogaster (Fruit Fly)*	19.5	NC-024511	-	-	-
		*Formica fusca (Black Ant)*	16.6	NC-026132	-	-	-
	Chnidarians	*Aurelia aurita*	16.9	NC-008446	-	-	-
		*Chrysopathes formosa*	18.4	NC-008411	-	1	-
		*Hydra oligactis*	16.3	NC-010214	-	-	-
		*Metridium senile*	17.4	NC-000933	-	2	-
	Mammals	*Mus musculus (Mouse)*	16.3	NC-005089	-	-	-
		*Pan troglodytes (Chimpanzee)*	16.6	NC-001643	-	-	-
		*Homo sapiens (Human)*	16.6	NC-012920	-	-	-
		*Homo sapiens neanderthalensis*	16.6	NC-011137	-	-	-
Protista		*Dictyostelium discoideum*	55.6	NC-000895	-	6	-
		*Monosiga brevicollis*	76.6	NC-004309	-	4	-

^*1^ A group I intron is found in the *cox1* gene in the mtDNA of some angiosperms [69]; ^*2^ Genome sequencing suggests that an intracellular parasitic α-proteobacterial, like Rickettsia, share a common ancestor with modern mitochondria [70]; ^*3^ A Putative editing site in *trnI-CAU* [71]; ^*4^ A, Angiosperms; B, Bryophyta; G, gymnosperms.
